# A new era of cystic fibrosis therapy with CFTR modulators

**DOI:** 10.36416/1806-3756/e20240405

**Published:** 2024-12-30

**Authors:** Laura Gomes Boabaid de Barros, Laura Menestrino Prestes, Maria Fernanda Gonçalves Meirelles Fernandes, Luiz Vicente Ribeiro Ferreira da Silva-Filho, Leonardo Araújo Pinto

**Affiliations:** 1. Grupo de Pesquisa em Epidemiologia e Genética das Doenças Respiratórias da Infância, Pontifícia Universidade Católica do Rio Grande do Sul - PUCRS - Porto Alegre (RS) Brasil.; 2. Instituto da Criança e do Adolescente, Hospital das Clínicas, Faculdade de Medicina, Universidade de São Paulo, São Paulo (SP) Brasil.; 3. Programa de Pós-Graduação em Medicina - Pediatria, Escola de Medicina, Pontifícia Universidade Católica do Rio Grande do Sul - PUCRS - Porto Alegre (RS) Brasil.

## INTRODUCTION

Cystic fibrosis (CF) is an autosomal recessive genetic disorder caused by mutations in the cystic fibrosis transmembrane conductance regulator (*CFTR*) gene.^1^ Its presentation in the epithelial cells of the body involves multiple systems, including the respiratory, digestive and reproductive systems. Individuals with this condition exhibit abnormal transport of chloride and bicarbonate in the secretory epithelia, resulting in thick and viscous secretions in the aforementioned systems.^2^ However, the progressive lung disease remains the leading cause of both morbidity and mortality in CF. We are currently experiencing an exciting and significant change in the scenario of CF treatment, which can be attributed to the introduction of CFTR modulators in the treatment of the condition. These modulators are a class of molecules that work by improving the intracellular processing and function of the defective CFTR protein.^2^ By improving the expression and function of the CFTR protein, these molecules act on the basic defect of the disease, a scenario never seen with several previous therapies.^1^ It is worth noting that not all individuals with CF can use these therapies, as their actions are specific for some type of defects of CFTR expression/function, and therefore the indications rely on specific variants of the CFTR gene. As they represent completely new therapeutic approaches, the effects on the course of the disease have yet to be fully determined; however, due to the significant improvement of symptoms and lung function in individuals using modulators, it is likely that the life expectancy of CF patients will be considerably increased.^2^

## A NEW ERA OF CF THERAPY WITH MODULATORS

The CFTR modulators are divided into two main groups: correctors and potentiators. The correctors act mainly in the endoplasmic reticulum, where they improve the structure and transport of the defective CFTR protein to the cell surface, ensuring that more CFTR protein reaches the appropriate site where it should work. An example is elexacaftor, indicated as a combination with another corrector (tezacaftor) for individuals with the *F508del* variant, the most common CF variant worldwide. The potentiators enhance the activity of CFTR proteins located on the cell surface by prolonging the open state of the channel, thereby allowing chloride to flow through the cell membrane more effectively. An example is ivacaftor, which increases the duration of CFTR channel opening to improve its functionality.^1^ ^,^ ^2^

The first CFTR modulator approved by the US Food and Drug Administration (FDA) in January of 2012 and by the Brazilian *Agência Nacional de Vigilância Sanitária* (ANVISA, National Health Surveillance Agency) in March of 2015 was Kalydeco^®^ (ivacaftor). This modulator is indicated for CF individuals with gating variants and was initially tested in individuals with the *G551D* variant, the most common gating variant in the USA.^1^ ^,^ ^2^ This medication has shown significant improvements in lung function, nutritional status, and respiratory symptoms reported by individuals with eligible variants. In the Brazilian CF population, the most common CFTR gating variant is *S549R*, which has been shown to be responsive to ivacaftor as well.^3^ While *F508del* variants do have a gating defect when expressed in the cell membrane, the use of ivacaftor alone was not effective to rescue its function, and there was a need to improve processing and trafficking of *F508del* mutants. Therefore, to treat individuals with *F508del* variants, there was a need for combinations of correctors and potentiators, aiming to tackle both the expression and function of the CFTR protein.^1^ ^-^ ^3^

Following initial attempts of using combined modulators with low to moderate clinical effects, such as Orkambi^®^ (lumacaftor and ivacaftor) and Symdeko^®^ (tezacaftor and ivacaftor), a significant step was given when researchers identified the need for a combination of two correctors to improve *F508del* variants expression.^3^ This finding resulted in Trikafta^®^, a medication combining two correctors (elexacaftor and tezacaftor), and one potentiator (ivacaftor). The effects of this combination can be noticed in diverse epithelial cells lining tubular organs affected by CF, including the lungs, gastrointestinal tract, and pancreas.^1^ ^,^ ^3^ The results of clinical trials of Trikafta^®^ for individuals with CF with at least one copy of the *F508del* variant were striking on several outcomes, such as lung function, rate of pulmonary exacerbations, sweat chloride concentrations, quality of life, nutrition, and glycemic status.^2^ ^,^ ^3^ More data is emerging on real world experiences with this medication, as well as expansion of age groups and eligible *CFTR* variants.^3^ Trikafta^®^ was approved by the FDA in the United States in October of 2019, while it was authorized by ANVISA in Brazil in 2022.^4^ ^,^ ^5^ Fortunately, in June of 2023, Trikafta^®^ was incorporated into the Brazilian Public Health Care System, making it available to CF individuals who have at least one copy of the *F508del* variant and are 6 years of age or older.^4^

[Table t1a]

**Chart 1 t1a:** Mechanism of action and *CFTR* variants approved by the US Food and Drug Administrations and the Brazilian National Health Surveillance Agency.

Modulators	Action Mechanism	FDA approved mutations	ANVISA approved mutations
Kalydeco® (Ivacaftor)	Potentiator, which allows the protein to remain open longer, facilitating greater ion passage.	Patients aged 1 month and older, who have one of the following CFTR mutations: 711+3A®G; 2789+5G®A; 3272-26A®G: 3849+10kbC®T; A120T; A234D; A349V; A455E; A1067T; D110E; D110H; D192G; D579G; D924N; D1152H; D1270N; E56K; E193K; E822K; E831X; F311del, F311L; F508C; F508C, S1251N†: F1052V; F1074L; G178E; G178R; G194R: G314E; G551D; G551S; G576A; G970D; G1069R; G1244E; G1249R; G1349D; H939R; H1375P: 1148T; 1175V; 1807M; 11027T; 11139V; K1060T; L206W; L320V; L967S; L997F; L1480P; M152V; M9521; M952T; P67L; Q237E; Q237H; Q359R; Q1291R; R74W: R75Q; R117C; R117G; R117H; R117L; R117P; R170H; R347H; R347L; R352Q; R553Q: R668C; R792G; R933G; R1070Q; R1070W; R1162L; R1283M; S549N; S549R; S589N; S737F: 5945L; 5977F; S1159F; S1159P; S1251N; S1255P; T3381; T10531; V232D; V5621; V754M: V1293G; W1282R; Y1014C; Y10320.	Patients aged 6 years and older, weighing 25 kg or more, who have one of the following gating (class III) CFTR mutations: G551D, G1244E, G1349D, G178R, G551S, S1251N, S1255P, S549N, or S549R. Also for patients aged 18 years and older who have an R117H mutation.
Trikafta® (Elexacaftor + Tezacaftor + Ivacaftor)	Correctors (Tezacaftor and Elexacaftor), which help more CFTR proteins reach the membrane, and a potentiator (Ivacaftor)	Patients aged 2 years and older, who have one of the following CFTR mutations: 3141del9; 546insCTA; A46D; A120T; A234D; A349V; A455E; A554E; A1006E; A1067T; D110E: D110H; D192G; D443Y; D443Y, G576A, R668C†; D579G; D614G; D836Y; D924N; D979V; D1152H; D1270N; E56K; E60K; E92K; E116K, E193K; E403D; E474K; E588V; E822K; F191V; F311del; F311L; F508C; F508C, S1251N† F508del; F575Y; F1016S; F1052V; F1074L; F1099L; G27R; G85E; G126D; G178E; G178R; G194R; G194V; G314E; G463V; G480C; G551D; G551S; G576A; G576A, R668C+; G622D; G628R; G970D; G1061R; G1069R; G1244E; G1249R; G1349D; H139R; H199Y; H939R; H1054D; H1085P: H1085R; H1375P: 1148T: 1175V; 1336K: 1502T: 1601F: 1618T: 1807M; 1980K; 11027T: 11139V; 11269N: 11366N; K1060T; L15P; L165S; L206W; L320V; L346P; L4535; L967S; L997F; L1077P; L1324P; L1335P; L1480P; M152V; M285R; M9521; M952T; M1101K; P5L; P67L; P205S; P574H; Q98R; Q237E; Q237H; Q359R; Q1291R; R31L; R74Q; R74W; R74W, D1270N; R74W, V201M; R74W, V201M, D1270N; R75Q; R117C; R117G; R117H; R117L; R117P; R170H; R258G; R334L; R334Q; R347H; R347L; R347P; R352Q; R352W; R553Q; R668C; R751L; R792G; R933G; R1066H; R1070Q; R1070W: R1162L; R1283M; R1283S; S13F; S341P: S364P; S492F; S549N; S549R; S589N; S737F; S912L; S945L; S977F; S1159F; S1159P; S1251N; S1255P; T3381; T1036N; T10531; V201M; V232D; V456A; V456F; V5621; V754M; V1153E; V1240G; V1293G; W361R; W1098C; W1282R; Y109N; Y161D; Y161S; Y563N; Y1014C; Y1032C.	Patients aged 6 years and older who have at least one F508del mutation in the CFTR gene.

## CONCLUSION

The management of CF has been changing drastically over the last few years, especially after the introduction of CFTR modulators. Currently, 188,336 people have the disease in 96 countries, but only 27% of them gained access to this type of treatment. These data indicate that although there is an increasing availability of CFTR modulators, a significant disparity in access to these therapies is noticed, mainly in countries with limited financial resources.^6^ The main barrier to a wider access remains the cost of these therapies. This sad situation needs a global collaboration toward new solutions to improve access, mainly in such countries. We hope that a true expansion of access to these transforming therapies will actually occur in the next few years, impacting the quality of life and life expectancy of individuals from different cultures and places of the world.
